# Genome and network visualization facilitates the analyses of the effects of drugs and mutations on protein-protein and drug-protein networks

**DOI:** 10.1186/s12859-016-0908-x

**Published:** 2016-03-02

**Authors:** Arnaud Céol, Lisette G. G. C. Verhoef, Mark Wade, Heiko Muller

**Affiliations:** Center for Genomic Science of IIT@SEMM, Fondazione Istituto Italiano di Tecnologia (IIT), Via Adamello 16, Milan, I-20139 Italy

**Keywords:** Protein networks, Structures, Genome variants, Genome browsing, Visualization, Protein-protein interaction, Protein-ligand interactions

## Abstract

**Background:**

Biologists generally interrogate genomics data using web-based genome browsers that have limited analytical potential. New generation genome browsers such as the Integrated Genome Browser (IGB) have largely overcome this limitation and permit customized analyses to be implemented using plugins. We illustrate the use of a plugin for IGB that exploits advanced visualization techniques to integrate the analysis of genomics data with network and structural approaches.

**Results:**

We show how visualization technologies that combine both genomics and network biology can facilitate the selection of the key amino acid contacts from protein-protein and protein-drug interactions. Starting from the MDM2-P53 interaction, which is a high-value target for cancer therapy, and Nutlin, the parent small molecule of an MDM2 antagonist that is currently in clinical trials, we show that this method can be generalized to analyze how drugs and mutations can interfere with both protein-protein and drug-protein networks. We illustrate this point by two additional use-cases exploring the molecular basis of tamoxifen side effects and of drug resistance in chronic myeloid leukemia patients.

**Conclusions:**

Combined network and structure biology approaches provide key insights into both the genetic and the edgetic roles of variants in diseases. 3D interactomes facilitate the identification of disease-relevant interactions that can then be specifically targeted by drugs. Recent advances in molecular interaction and structure visualization tools have greatly simplified the mapping of mutated residues to molecular interaction interfaces. Such approaches can now also be integrated with genome visualization tools to enable comparative analyses of interaction contacts.

**Electronic supplementary material:**

The online version of this article (doi:10.1186/s12859-016-0908-x) contains supplementary material, which is available to authorized users.

## Background

The wealth of available genomics data has created an analysis bottleneck; efficient means of candidate gene prioritization that permit the integration, comparison and interpretation of genome-scale molecular information are therefore urgently needed. In particular, network and structural biology criteria must be applied to candidate genes in order to pinpoint their contribution to the development of cancer-specific phenotypic traits and to evaluate their druggability. In general terms, the former can be evaluated by studying the connectivity of candidate genes with bona-fide cancer genes in protein-protein interaction (PPI) networks, while the latter can be deduced from structural information on the protein of interest.

Disease networks (networks of disorders and disease proteins) facilitate the identification of connections between disease-causing gene defects [1, 2] and mapping genomic regions to the networks of interactions with structures (3D networks) allows data-driven hypothesis generation about mutations that are likely to have edgetic effects [3], i.e. a loss or gain of interaction. Protein-ligand interactions (PLIs) can reveal valuable hints about the druggability of candidate genes as well as the contact regions of their binding partners. The development of drugs interfering with PPIs is challenging due to the nature of the PPI interface. The first successful example was the 1986 report of peptide-mediated inhibition of Herpes simplex type I ribonucleotide reductase [4, 5]. Since then, important progress has been made. It has been recognized that druggable PPIs largely fall into four different classes [6]. The first class employs short peptide epitopes that are bound by another protein. The second class is based on secondary structure epitopes where a single peptide from one protein binds to a groove presented by the binding partner. The major part of the binding energy is provided by a small number of amino acids called hotspots. The third class of PPIs utilizes large and shallow globular regions called tertiary structure epitopes. The fourth class is based on allosteric interactions. The second class of PPIs has sparked particular interest due to the presence of binding pockets and hotspot amino acids that can be identified by experimental and computational methods, and whose function can be disrupted by relatively small compounds developed using traditional medicinal chemistry [6]. It is reasonable to assume that hotspot amino acids are preferred targets of mutations in cancer and other diseases. Therefore, computational tools facilitating the mapping of mutations identified in genomic screens to protein structures are expected to be of considerable help in identifying hotspots.

Knowledge of the structures of proteins and PPIs is required to understand the mechanism of action of drugs and how they interfere with protein networks. The Protein Data Bank (PDB) [7] contains almost 30,000 structures that include human molecules. Those structures describe more than 4500 human direct interactions (estimation based on the number of experimental structures available in Interactome3D version 2015_05). Although the coverage of the protein network is far from being complete, high quality models such as those proposed by Interactome3D [8] have extended the human PPI network with models for 4294 additional human PPIs. It is important to note that all interactions in Interactome3D, including those based on a model only, are supported by experimental evidence (e.g., yeast two hybrid or co-immunoprecipitation), and that the modeling framework has been validated to ensure that only high quality models are provided.

Combining data on protein structures and genomic screens for integrated analysis is a non-trivial task. We have recently developed a plugin for IGB [9] that uses advanced visualization techniques to integrate the analysis of genomics data with network and structural biology approaches [10]. The plugin automatically maps genomic regions to protein sequence and interaction structures and identifies residues in contact with proteins, nucleic acids or small molecules. Here we show how such visualization technologies that combine both genomics and network biology can be used to map genomic variations to molecular networks, and to identify hotspots based on protein-protein and protein-drug interactions. This allows the end user to generate hypotheses regarding drug- and ligand-dependent perturbations of PPI networks, and provides predictions as to how specific mutations might have an impact on drug resistance.

## Results and discussion

### Protein-protein and protein-ligand interactions in structure databases

The PDB contains structures for 5387 human proteins from UniProt (02/2015) [11]. Those structures describe the coordinates of either a single molecule or of a complex that can include one or more binding partners (proteins, nucleic acids, drugs or other compounds). Based on PDB, Interactome3D has built a network of 8880 human interactions with structure (including experimental structures and models). Many of the proteins in this 3D PPI network also interact with drugs and other compounds. We compared the interfaces in PPI structures to the interfaces of protein-ligand interactions (PLIs). Our premise was that compounds that share one or more contact residues with a protein are potentially capable of interfering with the PPI.

**Fig. 1:**
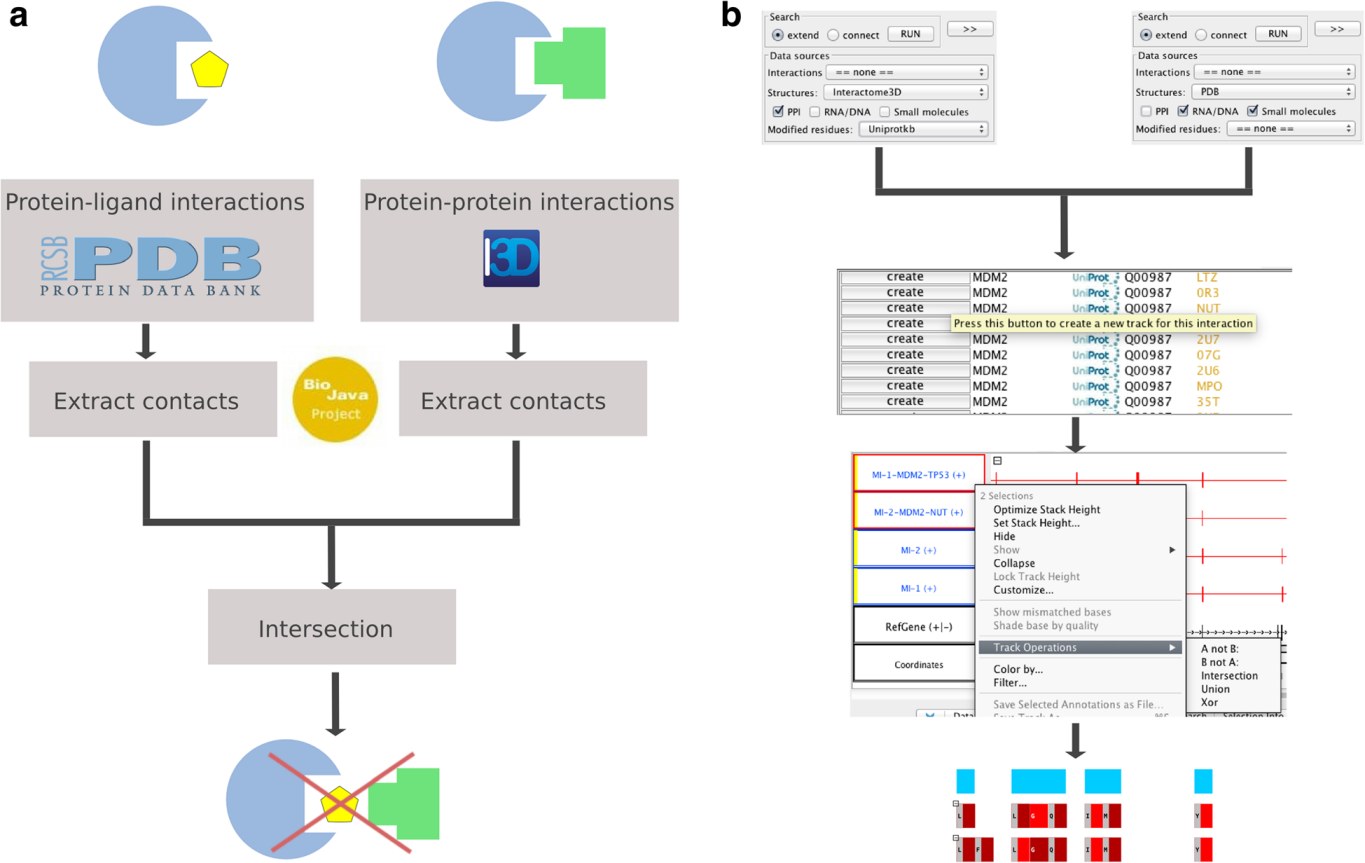
Pipeline for the comparison of protein-protein and protein-ligand analyses. **a** General pipeline: the structures for PLIs are downloaded from PDB and those for PPIs from Interactome3D. We use the BioJava library to extract protein-protein and protein-ligand contacts. Finally, we calculate the intersection between PPI and PLI contacts to identify the drugs that may interfere with the PPIs. **b** Visualization and analyses with IGB/MI-Bundle: we select a gene of interest in IGB and run the plugin twice: 1) on Interactome3D, with the PPI option, 2) on PDB, with the Small Molecules option. For each query, a result table is generated. We select the PPI and PLI interaction of interest in the respective table and for each of them we press the “create track” button. The generated tracks show all the residues of the selected protein that are in contact in a given interaction. We select both tracks and calculate the intersection (right click, then “track operations” and “Intersection”). IGB creates a new track where all residues of the selected protein that are in contact with the other protein and the small molecule are shown

We started by retrieving the list of structures from PDB that include a compound and extracted all contacts between proteins and ligands (Fig. 1a). Then, we inspected all human PPIs in the pre-calculated structural human network from Interactome3D, including models, to identify contacts in common with the ligands (residues with Buried Surface Area > 1 Å^2^, see methods). We identified 1253 ligands that bind at the interface of 1123 human binary PPIs (meaning that at least one contact is shared with PLI). The interactions are based on 473 complete experimental structures and 650 complete homology models (>80 % coverage) or partial experimental structures or models.

**Fig. 2:**
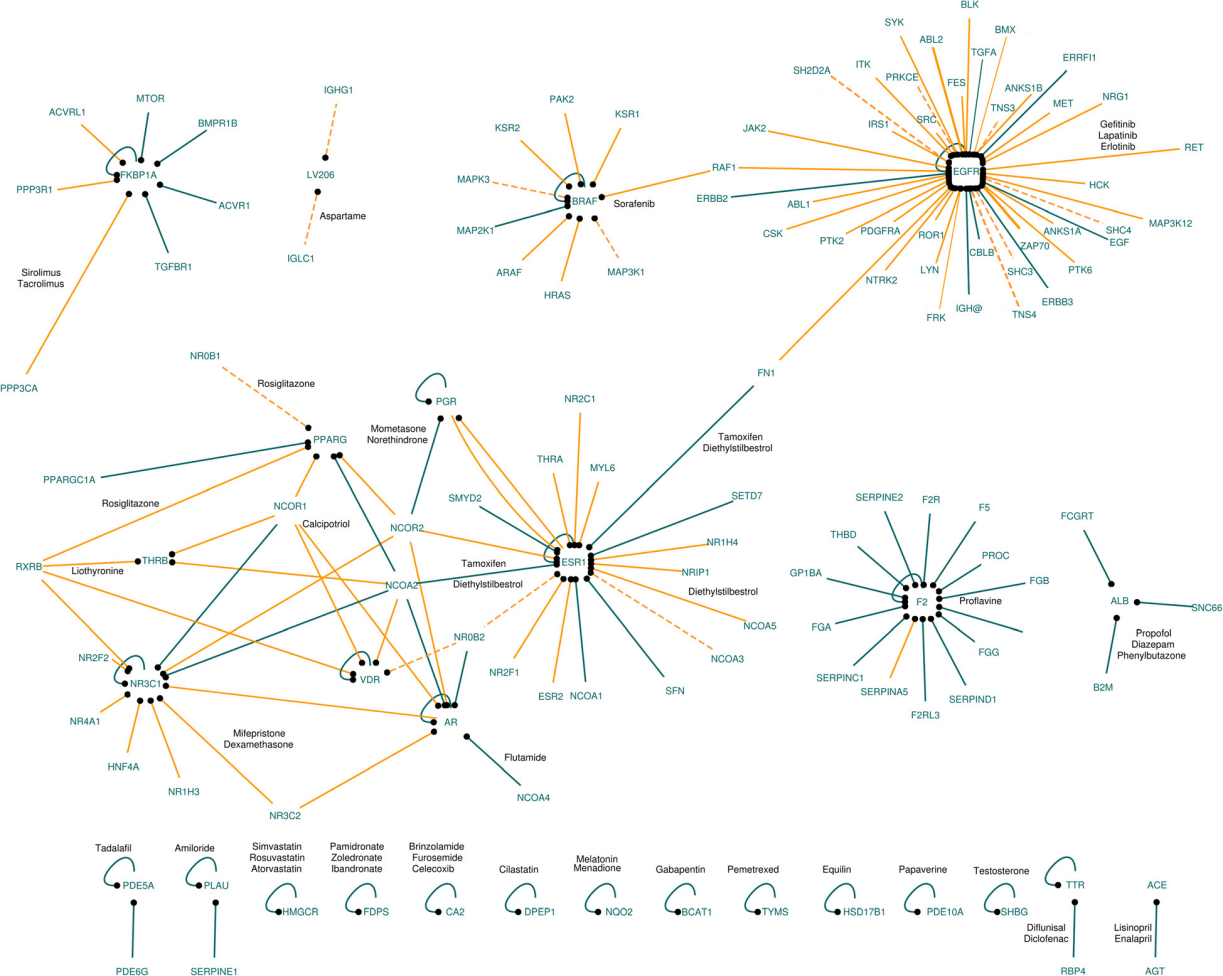
Network protein-protein interactions with drug binding interface. Network of PPIs whose interface is shared with a drug (from DrugBank). Dark edges: interactions with complete experimentally determined structure; Orange edges: interactions with complete homology models; dashed lines: interactions with partial experimental structures or models. Edge label: names of the drugs targeting the protein indicated with a small black circle. For clarity, the name of the drugs is only indicated on one edge for each target

Many of those ligands (for instance solvents, salts, molecules used for experimental purposes, and cofactors) may be irrelevant when looking for molecules that may alter the PPI network. Therefore, we restricted the list of ligands in PDB to include only small molecules approved by the U.S. Food and Drug Administration (FDA) listed in DrugBank [12] (see Methods). Of the 1253 interface compounds we identified above, 44 are approved small molecules that are cross-referenced in DrugBank. Those 44 drugs are at the interface of 151 PPIs (Fig. 2). None of those compounds were originally designed to target a PPI. This network contains several well-known drug targets, including cancer targets such as EGFR and BRAF. The amino acids in contact with the drugs are also in contact with many proteins involved in signal transduction (46 %, Gene Ontology enrichment *p*-value: 7e–19), in response to stimulus (85 %, *p*-value 3e–16), and they are mainly membrane proteins (72 %, *p*-value: 8e-7, see Additional file 1 for the complete list of enriched terms). Interestingly, many of those contacts have been identified in complete homology models (68 interactions) or partial experimental structures or models (14 interactions), rather than in complete experimental structures (68). Some interactions have been modeled from the homologs of the interacting proteins in other closely related species. For example, the NCOA2-VDR interaction (uniprotkb:Q15596 and uniprotkb:P11473) was modeled based on a structure that used the Zebrafish homolog of human VRD (PDB:3o1d). An experimental structure can also be used to model other interactions in the network. For instance, the model for the BRAF-RAF1 (uniprotkb:P15056 and uniprotkb:P04049) interaction is based on the experimental structure of the BRAF homodimer (PDB: 4ehe,4mbj). Since these results were able to pinpoint cancer-relevant pathways, and also predict the influence of small molecules on PPIs, we next used genome and network visualization to analyze some examples of drug interactions.

### Drugs in protein-protein networks: a visualization perspective

By intersecting the structural protein-protein and protein ligand networks above, we observed that many small molecules, including several approved drugs, could potentially compete with other proteins for binding at interaction sites. However, these analyses required programming skills, the download of datasets with diverse formats from external repositories, and basic knowledge of network and structural biology. To overcome these issues, and make the tool more accessible to a broader scientific user base, we have integrated the analysis pipeline into a single visualization tool as an IGB plugin called Molecular Interaction (MI) bundle [10]. Several improvements have been implemented and integrated (version 2.9 of the plugin), including the option of querying PDB for ligands only (in previous versions, PPIs were always included), and the integration of information from DrugBank (including drug name and ID) if a cross-reference is available.

Here we used this plugin to visually investigate several examples of drug-PPI interactions, and to integrate mutations and drug-ligand data in order to identify gatekeeper mutations (mutations that abrogate binding of drugs to their targets).

The analyses can be done in 5 steps (Fig. 1b):

1. Choose drug target and select the corresponding gene in IGB,
2. Select PDB and small molecules in the MI-Bundle to obtain a list of structures with ligands,
3. Select Interactome3D to obtain a list of PPIs with either a structure or a model,
4. Create a new track for each single interaction of interest: the PLI and the PPI by clicking on the “create track” button on the selected row in the result table,
5. Select tracks of interest and calculate the intersection using basic IGB functionalities.

We illustrate this pipeline with the examples of the p53-activating drug Nutlin, the estrogen receptor modulator Tamoxifen, and the development of resistance to Imatinib in chronic myeloid leukemia patients (see also Additional file 2: Additional example of drugs targeting PPIs).

### The MDM2-P53 druggable interaction, a case study

P53 is a transcription factor that mediates cell cycle arrest, senescence, or apoptosis in response to DNA damage or oncogenic stress. Consistent with its bona fide tumor suppressor status, the function of p53 or its regulators is altered in most cancers. The MDM2 oncoprotein (uniprotkb: Q00987) is overexpressed in a large fraction of human tumors, and exerts its activity primarily via inhibition of P53 (uniprotkb: P04637). The N terminus of P53 is required for its activity as a transcription factor; it is also the critical domain required for binding to MDM2 [13]. Once bound by MDM2, P53 transactivation function is inhibited. Furthermore, the intrinsic E3 ubiquitin ligase activity of MDM2 leads to proteasome-dependent P53 degradation. Thus, targeting of this interaction has received great attention, as it is expected to reactivate P53 in the ~50 % of tumors that retain a wild type allele [14].

**Fig. 3:**
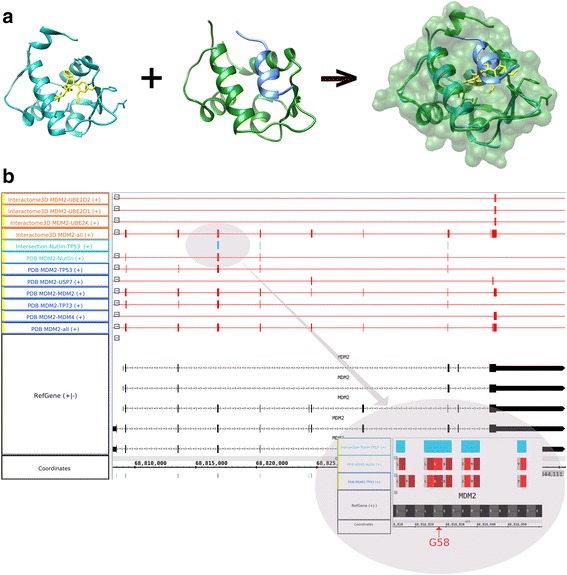
The small molecule Nutlin interferes with the MDM2-TP53 interaction. **a** Structure of Nutlin (yellow) binding to MDM2 (green), of the MDM2 (green)/P53 (blue) complex. The alignment of the structures shows that the P53 peptide and Nutlin interact with the same binding pocket. **b** Interaction contacts in the genome browser. The bottom track (black) displays the MDM2 transcripts. The other tracks display the contacts between MDM2 and one or more molecules. Dark blue tracks: protein-protein contacts from experimental structures. Green track: Nutlin-MDM2 contacts from experimental structure. Turquoise track: interaction between the Nutlin-MDM2 and the P53-MDM2 tracks. Orange tracks: contacts from models. A zoom on the intersection track evidences several MDM2 residues in contacts with both Nutlin and P53, including G58

Starting our pipeline from the *MDM2* gene (Fig. 3), we pinpointed in the MDM2/P53 track several residues important for the binding between those two molecules; A cell-based bimolecular luciferase complementation (BiLC) assay indicates an effect of the mutation of the G58 residue on the PPI (see Additional file 2: Selection of the key amino acid contacts from protein-protein and protein-drug interactions for experimental manipulation). Other residues (e.g. V75) had already been reported in literature [15].

In the list of partners of MDM2, we found the cis-imidazoline, Nutlin (NUT, CHEBI:46,742), which was identified during screening of a compound diversity set performed by Roche [16]. The residues on the intersection track (MDM2/P53 and MDM2/Nutlin) are the MDM2 residues that Nutlin shields from p53. These residues are Phe19, Trp23, and Leu26, which are located in an alpha-helical region of the P53 N terminus that binds to the N-terminal MDM2 hydrophobic pocket [17]. The imidazoline scaffold of Nutlin essentially mimics these three critical P53 residues; the compound therefore competes with endogenous P53 for binding to MDM2. In the absence of a structure between MDM2 and P53 and knowing that Nutlin disrupts this interaction, it would have been possible to exploit our strategy to infer some of the contact residues between MDM2 and P53.

MDM2 is involved in three additional interactions for which a structure is available. We created a new track to display the contacts with each of those: MDM2, USP7 (uniprotkb:Q93009) and MDM4 (uniprotkb:O15151). Interestingly, the MDM2 homo-dimerization site intersects with the MDM2-Nutlin interface, suggesting that Nutlin may also interfere with MDM2 homodimerization. Conversely, the contacts that MDM2 makes with USP7 and MDM4 are distinct from the ones with Nutlin: The MDM2/USP7 and MDM2/MDM4 interactions may not be affected by this ligand, suggesting an edgetic effect of this compound. Our prediction that Nutlin does not interfere with the MDM2/MDM4 interaction is supported by data showing that MDM2 and MDM4 co-immunoprecipitate following Nutlin treatment, which is consistent with Nutlin-stimulated, MDM2-dependant degradation of MDM4 [18, 19].

In order to determine whether Nutlin might perturb other interactions in which MDM2 participates, we repeated the previous query with Interactome3D resulting in the extension of the 3D coverage of the MDM2 network with models for interactions with three E2 enzymes (Ubiquitin-conjugating enzyme E2), UBE2D1 (uniprotkb:P51668), UBE2D2 (uniprotkb:P62837), and UBE2K (uniprotkb:P61086). Based on the models, none of those interactions share contact regions with Nutlin; those models nevertheless confirm the importance of the C-terminal MDM2 RING domain in mediating its E3 ubiquitin ligase activity [20]. It is worth noting that the contacts are based on structures and models that are not complete, and that additional interfaces may exist.

### Another mechanism for tamoxifen action

**Fig. 4:**
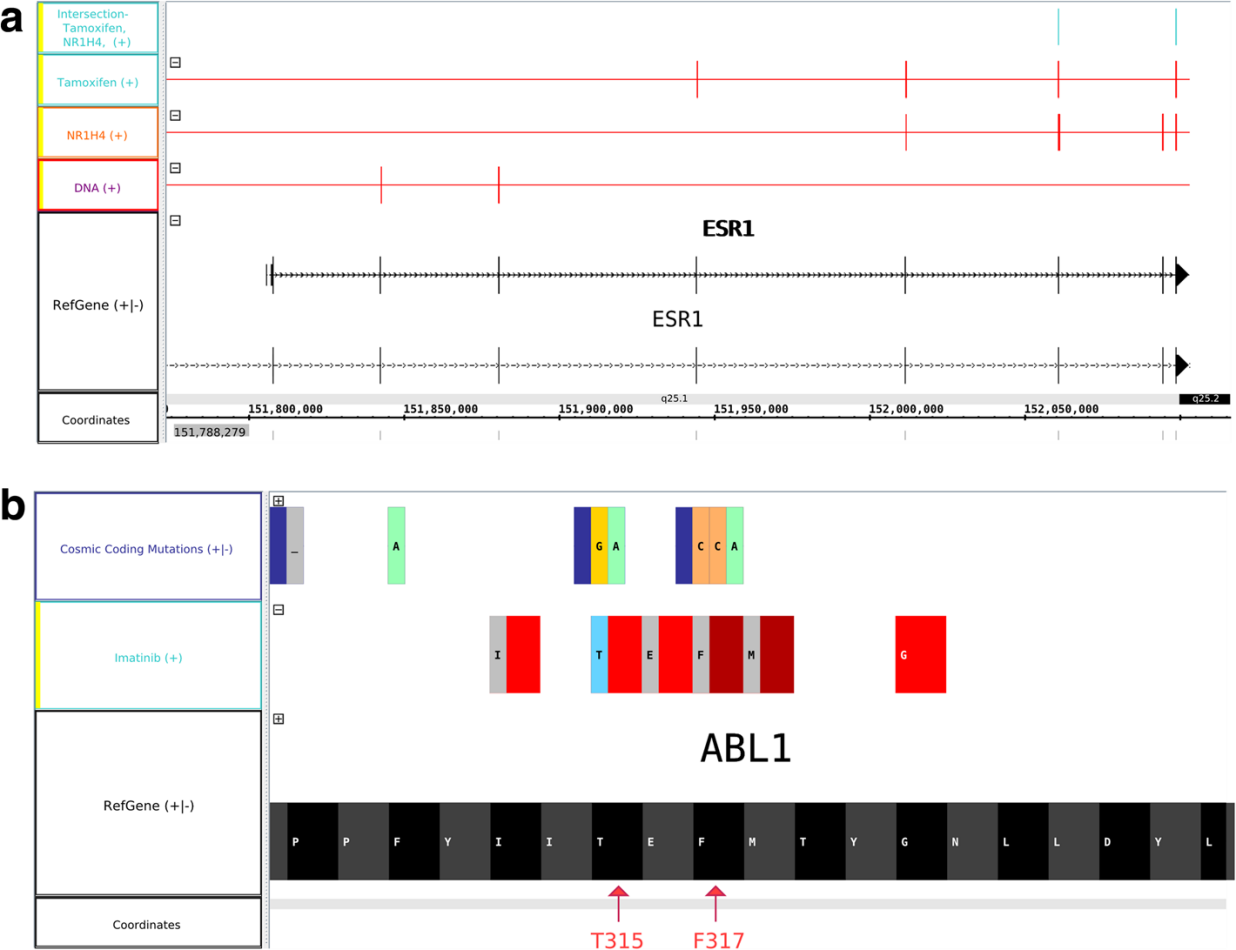
Examples of contact comparison analyses. **a** Three residues of ESR1 (Asp426, Met522 and Pro535) are involved in the binding with both Tamoxifen and NR1H4. **b** Cancer somatic mutations of ABL1-Imatinib contact residues. T315 and F317 are two examples of gatekeeper mutations involved in drug resistance

Intriguingly, our modeling suggests a novel PPI target for Tamoxifen, a small molecule that is used in the clinic to treat estrogen-dependent breast cancer [21]. Tamoxifen’s main mechanism-of-action is to compete with estrogen for binding to the estrogen receptor (ESR1), thereby preventing the pro-survival and proliferative effects of this hormone [22]. In addition to steroid hormones, ESR1 has several protein-binding partners, including NR1H4 (Fig. 2), a nuclear receptor that regulates the metabolism of cholesterol, lipid, and glucose. We observed that both Tamoxifen and NR1H4 share a common binding interface with ESR1 (Fig. 4a). Following the steps outlined above, an intersection track confirmed that Tamoxifen and NR1H4 have common contact residues with ESR1. Thus, Tamoxifen might antagonize the interaction between ESR1 and NR1H4. This could have functional consequences, since NR1H4-dependent growth of tumor cell lines in vitro is dependent on the ESR1/NR1H4 interaction [23].

### Mutations inducing drug resistance: the case of imatinib

Although many effective anticancer drugs have been developed, advances are often hampered by the development of drug resistance. There are several mechanisms that cause drug resistance, including target cell extrinsic mechanisms, tumor microenvironment, and gene polymorphisms that affect gene expression, for instance [24]. Gatekeeper mutations are defined as those that abrogate binding of drugs to their targets, dramatically reducing the clinical efficacy of treatment. A classical gatekeeper mutation causes resistance to Imatinib, a successful ATP-competitive BCR-ABL kinase inhibitor.

A search for ligands of ABL1 in PDB indicates that the structure of this protein in contact with Imatinib has been resolved (PDB: 2hyy and 3pyy). A new track can be created to display the residues that are actually in contact. We also downloaded from COSMIC [25] a list of missense mutations and loaded them into the browser. The comparison of mutated and contact residues identified several common mutations, which indeed do engender resistance to Imatinib (T315I and F317L) [26]. Importing mutation data in IGB is an easy task. In the future, in combination with the MI-Bundle, it will be possible to submit any newly discovered mutation to PLI analyses to identify new gatekeeper mutations.

## Conclusions

Genomics and molecular analysis tools are now widely available and sufficiently user friendly to allow cross-disciplinary investigation of the impact of genomic variants on biological systems. In this manuscript, we have used an extension for the IGB to illustrate the identification of key residues for the binding of MDM2 to P53, of Tamoxifen to NR1H4, and of gatekeeper mutations inducing resistance to Imatinib. Our IGB plugin greatly simplifies the integration of genomics data with network and structural analyses of both protein-protein and PLIs.

The particular example of the MDM2-P53 interaction is derived from the intersection of 3D protein-protein and protein-ligand networks. Nevertheless, the structural coverage of the protein network is largely incomplete. The compound-binding proteins we have identified in this work are involved in many interactions for which no structure is available. Thus, we are likely missing additional interactions that may be targeted by small molecules. However, it is possible to increase the structural coverage of the protein network with Interactome3D, which includes PPI models for interaction supported by experimental evidence. Although indirect interactions (e.g., in large complexes detected by co-immunoprecipitation) are excluded from the underlying experimental PPI network in Interactome3D, false positives may still limit the accuracy of the pipeline. The score implemented by the MI bundle may help to identify the most reliable interactions.

By looking at the intersection between protein-ligand contacts and protein-protein contacts, we were able to identify small molecules that may interfere with PPIs. To be clear: our aim with this modeling approach is not to diminish the importance of the conventional approaches to discover the mode of action of those drugs. However, we suggest that our integrated tool can be exploited to generate new hypotheses. It may also be useful for the identification of drugs or molecular scaffolds that can be repurposed in order to block PPIs.

Here, we focused on approved small molecules. Nevertheless, many other ligands have been crystalized together with target proteins and deposited in PDB, including experimental molecules and biotech drugs (peptide, protein or nucleic acid drugs). By extending the analyses to these additional compounds, we believe that it will be possible to generate predictions regarding their effects on disease-relevant PPI networks.

Our analyses are restricted to human proteins. It will be interesting to extend it to other species, both model organisms (many structures are generated based on the homologs of human proteins), and in host-pathogen interactions. To this end it may be useful to model not only PPIs, but also PLIs.

For our initial validation and testing, we have focused our analyses solely on contact residues, considering that one contact may be enough to imply a steric clash when the two partners are superimposed. We didn’t assess the extent of cases in which the protein structure of a PLI includes a protein interface that is significantly different from the interface between the same protein and another protein (PPI). We would increase the accuracy of the method by checking the number of clashing atoms. Indeed, in the examples based on the MI-bundle, we were able to visually verify that the respective proteins and drugs share several residues. This current limitation will also be restrained by extending the intersection analysis to the entire interaction interface. Employment of other tools and databases may be useful in this regard [27, 28]. In addition, it will be interesting to discriminate biological interfaces from crystal artifacts. This can be done for instance with the EPPIC server [28] (see Additional file 2: Selection of the key amino acid contacts from protein-protein and protein-drug interactions for experimental manipulation).

We have shown that all our analyses can be done without prior bioinformatics skills by using IGB together with the MI bundle. The software allows loading, visualization, comparison, and analysis of protein-protein and PLIs. Importantly, such analyses are not limited to the current knowledge deposited in public databases. The MI-bundle allows a local copy of an Interactome3D directory to be used. Any user that generates new protein networks (e.g. by yeast two hybrid) can submit them to the Interactome3D server and download the resulting structural network. Consequently, the user will be able to apply the plugin to their own data to discover new putative drug-PPI interactions.

As a genome browser, IGB accepts a large number of genomic formats, such as BED, VCF or GTF; it can also communicate directly with DAS servers [29], such as those implemented by the UCSC Genome Browser [30, 31] or Ensembl [32]. Therefore, it is easy to interrogate newly discovered mutations for their potential to induce drug resistance (gatekeeper mutations).

Finally, we did not consider the features of PPIs that may influence the success of an inhibitor in this manuscript. In a recent review, Smith and Gestwicki [6] have analyzed how amenable each of the four PPI classes are to inhibition, according to whether the interface is classified as “Loose” or “Tight”, and “Wide” or "Narrow". Integrating this information in our frameworks will facilitate the identification of the most promising PPI targets.

## Methods

### Drugs and compounds

We used the mapping file available at [33] to associate the compounds in the structures to DrugBank IDs (DrugBank version 4.2) [12]. This file only contains approved drugs. We downloaded the file [34] to obtain the list of known targets from DrugBank.

We used the mapping between Uniprot and PDB provided by the SIFTS initiative [35].

### Structures

We worked on a local mirror of the PDB database updated on May 2015. For PPIs, we downloaded the representative human dataset from Interactome3D, version 2015_05. The structures in Fig. 3 have been aligned and displayed with the UCSF Chimera package [36].

### Contacts

In order to identify the contacts between the pairs of proteins or the pairs of protein-compound identified in the previous step, we measure the available buried surface area as described in [10]. We consider as contact all residues with Buried Surface Area > 1 Å^2^. Parsing of PDB files and calculation of the available surface area was done with the BioJava libraries version 4.1 [37].

### Mutation data

We have downloaded the VCF file of all coding mutations in Cosmic [25] version 4.1 for the human reference genome version GRCh38 from [38].

### Network visualization

The network figures have been generated with Cytoscape version 3.2.0 [39].

### Gene ontology enrichment

We used the BINGO plugin [40] for Cytoscape to obtain GO enrichments on the list of partners of the drug targets. To avoid a bias toward proteins with structures, we used as reference set all the human proteins included in the human 3D network of Interactome3D. We downloaded from GOA [41] the GO annotations for human (generated: 2015-07-20). We used the GOA slim ontology in order to obtain a smaller list of GO terms that covers all GO categories (Biological Processes, Molecular Function, Cellular Compartment).

### Analyses with IGB and the MI-bundle

We downloaded and installed IGB and the MI-Bundle as described at [42]. All analyses in IGB are done on the human reference genome version GRCh38.

## Additional files

Additional file 1:
**Additional table: Gene Ontology terms enriched in the partners of drug targets.** (PDF 58 kb) Additional file 2:
**Additional results: Selection of the key amino acid contacts from protein-protein and protein-drug interactions for experimental manipulation; Additional example of drugs targeting PPIs.** (PDF 499 kb)

## Abbreviations

FDA: the Food and Drug Administration
IGB: integrated genome browser
MI: molecular interaction
PDB: the Protein Data Bank
PLI: protein-ligand interaction
PPI: protein-protein interaction
