# Supplementary material for: Genome and network visualization facilitates the analyses of the effects of drugs and mutations on protein-protein and drug-protein networks
Source: BMC Bioinformatics. 2016 Mar 2;17(Suppl 4):54. doi: 10.1186/s12859-016-0908-x (PMC4896239; doi:10.1186/s12859-016-0908-x)
Supplement: Additional file 1: — Additional table: Gene Ontology terms enriched in the partners of drug targets. (PDF 58 kb) [file 12859_2016_908_MOESM1_ESM.pdf]

Gene Ontology terms enriched in the partners of drug targets

GO-ID	Description	p-value	corr p-value	Cluster frequency
4871	signal transducer activity	1.35E-16	7.13E-15	46%
50896	response to stimulus	1.23E-13	3.26E-12	85%
16301	kinase activity	2.42E-09	3.57E-08	31%
32501	multicellular organismal process	2.69E-09	3.57E-08	78%
4872	receptor activity	2.08E-08	2.20E-07	29%
16740	transferase activity	2.57E-07	2.27E-06	38%
7275	multicellular organismal development	3.04E-06	2.30E-05	64%
6928	cellular component movement	2.46E-05	1.63E-04	31%
30154	cell differentiation	7.12E-05	4.19E-04	50%
16020	membrane	1.46E-03	7.76E-03	72%
50789	regulation of biological process	1.99E-02	9.59E-02	90%
43170	macromolecule metabolic process	7.09E-02	3.13E-01	73%
5615	extracellular space	1.86E-01	7.59E-01	23%
7610	behavior	2.77E-01	1.05E+00	18%
8152	metabolic process	1.07E+00	3.73E+00	83%
43062	extracellular structure organization	1.12E+00	3.73E+00	12%
30528	transcription regulator activity	1.00E+01	3.07E+01	19%
51704	multi-organism process	1.04E+01	3.07E+01	19%
5488	binding	2.78E+01	7.76E+01	99%
3824	catalytic activity	3.25E+01	8.60E+01	52%
5515	protein binding	4.06E+01	1.02E+02	93%
9987	cellular process	6.10E+01	1.47E+02	96%
46903	secretion	8.41E+01	1.94E+02	10%
9986	cell surface	8.92E+01	1.97E+02	12%
7154	cell communication	1.17E+02	2.48E+02	18%
30234	enzyme regulator activity	1.99E+02	4.05E+02	15%
