# Supplementary material for: Genome and network visualization facilitates the analyses of the effects of drugs and mutations on protein-protein and drug-protein networks
Source: BMC Bioinformatics. 2016 Mar 2;17(Suppl 4):54. doi: 10.1186/s12859-016-0908-x (PMC4896239; doi:10.1186/s12859-016-0908-x)
Supplement: Additional file 2: — Additional results: Selection of the key amino acid contacts from protein-protein and protein-drug interactions for experimental manipulation; Additional example of drugs targeting PPIs. (PDF 499 kb) [file 12859_2016_908_MOESM2_ESM.pdf]

Genome and network visualization facilitates the analyses of the effects of drugs and mutations on protein-protein and drug-protein networks.

Arnaud Ceol, Lisette G.G.C. Verhoef, Mark Wade, Heiko Muller

Additional results

Results

Selection of the key amino acid contacts from protein-protein and protein-drug interactions for experimental manipulation

We used IGB in combination with the MI Bundle to identify contact residues at the MDM2/P53 interface. David and Sternberg [1] have recently suggested that that disease-causing mutations are preferentially located within the interface core (solvent inaccessible upon PPI, as opposed to interface "rim" residues that remain partially solvent accessible). We therefore used the EPPIC webserver to refine the list of contacts identified, and conserved only those at the core of a biological interface [2]. We identified 31 MDM2 residues in contact with P53, of which 16 were also in contact with Nutlin. EPPIC classifies 8 of them as core residues of a biological interface (L54, L57, G58, I61, V75, V93, I99 and L224).

We decided to validate our strategy by assessing experimentally whether one of those residues (G58) has indeed an effect on the affinity between MDM2 and P53. In order to address this, we made use of a cell-based bimolecular luciferase complementation (BiLC) assay [3]. Briefly, the N-terminal fragment

of MDM2 is fused to a C-terminal fragment of firefly luciferase, while P53 is fused to an N-terminal fragment of luciferase. Only when MDM2 and P53 interact is luciferase enzymatic activity reconstituted (Supplementary Figure 1a). Thus, interaction between this protein pair can be measured in a multi-well plate format using standard laboratory reagents and equipment (see Supplementary Methods). We introduced P53 and either $MDM2^{WT}$ or the $MDM2^{G58A}$ mutant, and compared luciferase signals in each case. As a negative control, we co-transfected BiLC constructs of P53 and the RING domain of MDM2; these fragments do not interact with one another. As expected, whereas the p53/$MDM2^{WT}$ pair (Supplementary Figure 1b, first bar) gave robust luminescence, there was no luminescence signal from the p53/MDM2 RING pair (Supplementary Figure 1b, third bar). Importantly, and consistent with our computational prediction, the signal from p53/$MDM2^{G58A}$ was much lower than p53/$MDM2^{WT}$ (Supplementary Figure 1b, second bar).

From our experimental method, we cannot decisively prove that G58 is indeed a critical residue, and indeed it will be interesting to examine other interface residues were more 'conservative' mutations are introduced. Although the interface residues may be re-arranged by G58A, additional experimental data indicates that such rearrangements are likely to be limited: This is since G58A significantly (but not completely) abrogates binding to p53 (Supplementary Figure 1b). Furthermore, the G58A mutant is still responsive to Nutlin 3A (data not shown). Together, these data indicate that G58A may be less disruptive than some other recently published interface mutations that are completely refractive to Nutlin 3A, such as Q24R and M62V [4].

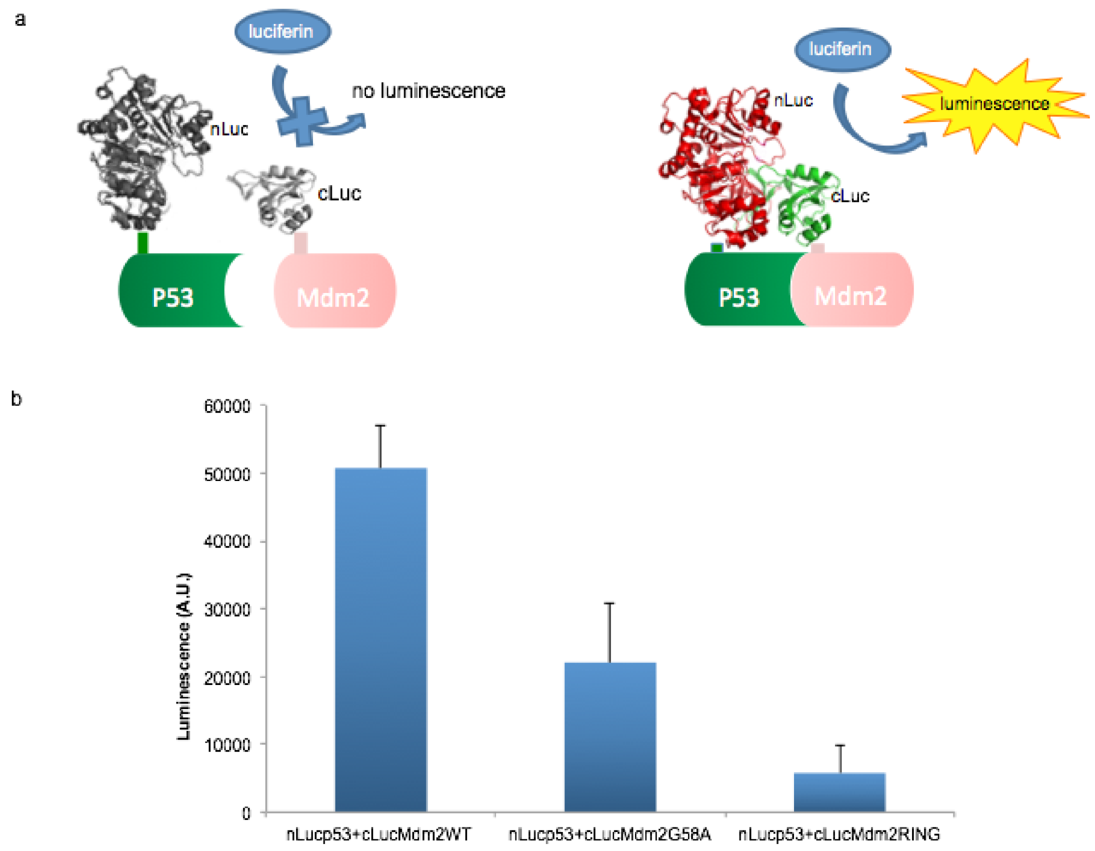

Supplementary Figure 1: The MDM2^{G58A} mutation disrupts binding to p53

a) Schematic representation of the BiLC strategy to measure PPI. An N-terminal fragment of firefly luciferase (nLuc) is fused to p53, and a C-terminal fragment (cLuc) is fused to MDM2. When p53 and MDM2 interact, the complementary parts of the firefly luciferase refold and its enzymatic activity is reconstituted. If the interaction is inhibited (either by mutagenesis, or with a small compound inhibitor such as Nutlin, for example) the enzymatic activity is reduced, resulting in a decrease in luminescence.

b) Quantitation of the interaction between P53 and MDM2^{WT}, MDM2^{G58A} or MDM2^{RING} measured by BiLC. U2OS cells were transfected with the indicated plasmids. Forty-eight hours post-transfection, luminescence (A.U.) was

measured. Mean ±SD of three independent experiments.

Additional example of drugs targeting PPIs

Tacromilus interferes with the ALK-1 / FKBP1A interaction

Hereditary hemorrhagic telangiectasia (HHT), is a genetic disorder that results in abnormal blood vessel formation. It is caused by mutations in the *ACVRL1*, *ENG*, and *SMAD4* genes. Both *ENG* and *ACVRL1* mutations lead predominantly to underproduction of the related proteins, rather than misfunctioning of the proteins [5].

In figure 2, we identify an interaction between ACVRL1 (uniprotkb:P37023, also named ALK1) and FKBP-1A (uniprotkb:P62942, also named FKBP-12).

A query in the MI-bundle (options: PDB, Small molecules) for the FKBP1A gene reports many ligands including two drugs: Sirolimus (Drugbank:DB00864) and Tacrolimus (Drugbank:DB:00877). There is a clear intersection between the residues from FKBP1A in contact with Tacrolimus and those in contact with ACVRL1 (MI-Bundle, option: Interactome3D, PPI). The model for the FKBP1A/ACVRL1 interaction is built upon the experimental structures of the FKBP1A/ACVR1 (uniprotkb:Q04771, PDB: 3h9r) and FKBP1A/TGFR1 (uniprotkb:P36897, PDB:1b6c) interactions. This provides a structural bases for the observation from Albinana et al. [6] that the amount of ACVRL1 pulled down by GST-FKBP1A was decreased when cells were treated with Tacrolimus. By interfering with the binding of ACVRL1, Tacrolimus may increase ACVRL1 activity, and eventually improve the symptoms of HHT.

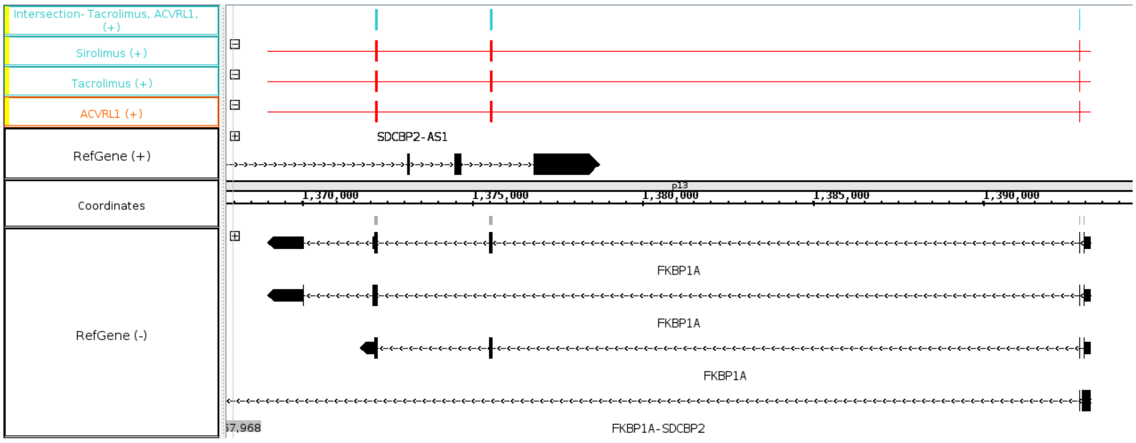

Supplementary figure 2: Tacrolimus and the ALK-1 / FKBP1A interaction

Experimental small molecules inducing conformational changes that interfere with the IL-2/IL-2RA interaction

In Figure 2, we show interfaces of PPIs that are targeted by approved drugs. In a recent review, Arkin et al. listed several ligands that are known to interfere with protein interactions. One of those is an experimental small molecule (SP4206) that disrupts the interaction between the inflammation-related cytokine, IL-2, and its cognate receptor, IL2RA.

We confirmed this mechanism-of-action of the ligand in IGB by searching for IL-2-related PLIs in PDB and PPIs in Interactome3D. As expected, IL2RA was identified as an IL-2 binding partner. SP4206 has been crystallized together with IL-2 under the name FRH (PDB:1PY2). Consistent with the experimental data, IL2RA and SP4206 have several contacts with IL-2 in common. Interestingly, the small molecule induces conformational changes, rather than directly competing for the protein-protein interface. In the case of the IL-

2/IL2RA interaction, this conformational change likely occurs at the binding site, making it inaccessible to the binding protein.

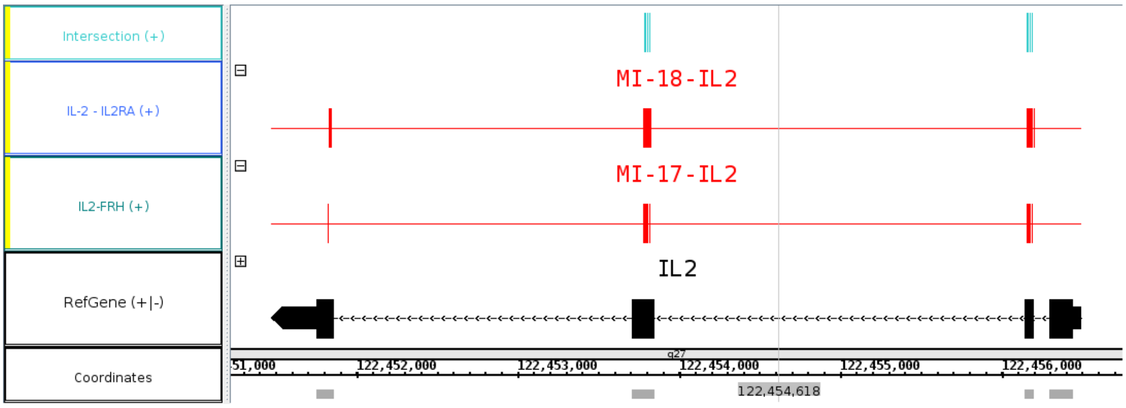

Supplementary figure 3: disrupting the IL-2/IL-2RA interaction

Methods:

Site directed mutagenesis, transfections and Bimolecular Luciferase Complementation (BiLC):

The $Mdm2^{G58A}$ mutant was made by performing site directed mutagenesis on the pcDNA3-$cLucMdm2^{WT}$ plasmid, which expresses the N-terminal 108 amino acids of Mdm2 and a C-terminal fragment of firefly luciferase (Agilent Technologies, QuikChange Lightning Site-Directed Mutagenesis Kit, 210518). The plasmid was sequenced to verify that the correct mutation was made.

The human osteosarcoma cell line U2OS was cultured using Dulbecco's Modified Eagle's Medium (Lonza, BE12-164F) supplemented with 10% FBS-NA (Euroclone, CHA30088L), and 2 mM L-glutamine (Lonza, 17-605E). Cells were transiently transfected with the indicated plasmids using a standard calcium phosphate protocol. Twenty-four hours post-transfection, cells were seeded into a 96-well plate (Corning Inc., Costar 3917), at a density of 5000 cells/well in DMEM/F12 without phenol red (Sigma-Aldrich, D6434). Forty-eight hours post-transfection, plates were equilibrated for 30 min at RT, an equal volume of Bright-Glo (Promega, Bright-Glo Luciferase Assay System E2620) was added, and luminescence was measured with a microplate reader (PHERAstar FS, BMG Labtech). Luminescence was corrected for cell viability by measurement of metabolic activity through the addition of an equal volume of resazurin, at a final concentration of 50 µM (Sigma-Aldrich, resazurin sodium salt, R7017). Cells were incubated for 2 h at 37°C, equilibrated for 30 min at RT, and fluorescence was measured using a microplate reader (PHERAstar FS, BMG Labtech).

References

1. David A, Sternberg MJE: The Contribution of Missense Mutations in Core and Rim Residues of Protein–Protein Interfaces to Human Disease. *J Mol Biol* 2015, 427:2886–2898.

2. Duarte JM, Srebniak A, Schärer M a, Capitani G: Protein interface classification by evolutionary analysis. *BMC Bioinformatics* 2012, 13:334.

3. Li Y, Rodewald LW, Wertman KF, Wahl GM, Li Y, Rodewald LW, Hoppmann C, Wong ET, Lebreton S, Safar P: Resource A Versatile Platform to Analyze Low-Affinity and Transient Protein-Protein Interactions in Living Cells in Real Time Resource A Versatile Platform to Analyze Low-Affinity and Transient Protein-Protein Interactions in Living Cells in Real Time. *CellReports* 2014, 9:1946–1958.

4. Wei SJ, Joseph T, Sim AYL, Yurlova L, Zolghadr K, Lane D, Verma C, Ghadessy F: In Vitro Selection of Mutant HDM2 Resistant to Nutlin Inhibition. *PLoS One* 2013, 8:1–14.

5. Abdalla SA, Letarte M: Hereditary haemorrhagic telangiectasia: current views on genetics and mechanisms of disease. *J Med Genet* 2006, 43:97–110.

6. Albiñana V, Sanz-Rodríguez F, Recio-Poveda L, Bernabéu C, Botella LM: Immunosuppressor FK506 increases endoglin and activin receptor-like kinase 1 expression and modulates transforming growth factor-β1 signaling in endothelial cells. *Mol Pharmacol* 2011, 79:833–843.
